# Generation and Functional Characterization of PLAP CAR-T Cells against Cervical Cancer Cells

**DOI:** 10.3390/biom12091296

**Published:** 2022-09-14

**Authors:** Vahid Yekehfallah, Saghar Pahlavanneshan, Ali Sayadmanesh, Zahra Momtahan, Bin Ma, Mohsen Basiri

**Affiliations:** 1 School of Biomedical Engineering, Shanghai Jiao Tong University, Shanghai 200030, China; 2 Department of Stem Cells and Developmental Biology, Cell Science Research Center, Royan Institute for Stem Cell Biology and Technology, ACECR, Tehran 1665666311, Iran; 3 Medical Nanotechnology and Tissue Engineering Research Center, Shahid Beheshti University of Medical Sciences, Tehran 1968917313, Iran; 4 Department of Applied Cell Sciences, Faculty of Advanced Medical Sciences, Tabriz University of Medical Sciences, Tabriz 5166653431, Iran; 5 Department of Medical Laboratory Sciences, Faculty of Allied Medical Sciences, Tehran Medical Sciences, Islamic Azad University, Tehran 1916893813, Iran; 6 Clinical Stem Cell Research Center, Renji Hospital, Shanghai Jiao Tong University School of Medicine, Shanghai 200127, China

**Keywords:** cervical cancer, chimeric antigen receptor, genetic engineering, T-cell therapy

## Abstract

Chimeric antigen receptor (CAR) T-cell therapy is one of the cancer treatment modalities that has recently shown promising results in treating hematopoietic malignancies. However, one of the obstacles that need to be addressed in solid tumors is the on-target and off-tumor cytotoxicity due to the lack of specific tumor antigens with low expression in healthy cells. Placental alkaline phosphatase (PLAP) is a shared placenta- and tumor-associated antigen (TAA) that is expressed in ovarian, cervical, colorectal, and prostate cancers and is negligible in normal cells. In this study, we constructed second-generation CAR T cells with a fully human scFv against PLAP antigen andthen evaluated the characteristics of PLAP CAR T cells in terms of tonic signaling and differentiation in comparison with ΔPLAP CAR T cells and CD19 CAR T cells. In addition, by co-culturing PLAP CAR T cells with HeLa and CaSki cells, we analyzed the tumor-killing functions and the secretion of anti-tumor molecules. Results showed that PLAP CAR T cells not only proliferated during co-culture with cancer cells but also eliminated them in vitro. We also observed increased secretion of IL-2, granzyme A, and IFN-γ by PLAP CAR T cells upon exposure to the target cells. In conclusion, PLAP CAR T cells are potential candidates for further investigation in cervical cancer and, potentially, other solid tumors.

## 1. Introduction

Immune cells can detect cancer cells and eliminate them by patrolling an individual’s body, but in the case of a cancer outbreak, immune cells’ anti-tumor effects do not have enough capacity to recognize and overcome cancer cells [1]. Hence, using modalities to enhance the potential of immune cells is imperative. For this goal, one of the methods that has shown remarkable results in cancer immunotherapy is chimeric antigen receptor (CAR) T-cell therapy. A typical second-generation CAR molecule contains an extracellular antigen-binding domain, usually a single chain fragment variable (scFv), linked to a transmembrane domain through a hinge and/or spacer domains, and intracellular signaling domains from CD3 subunits and costimulatory molecules such as CD28 and 4-1BB [2]. This structure allows T cells to recognize a broad range of cell surface antigens in an HLA-independent manner [3].

CAR T cells have demonstrated remarkable results in hematopoietic malignancies [4,5]. Especially, in a recent study by Melenhorst et al., they found that CD19-specific CAR T cells not only have anti-tumor capabilities against chronic lymphocytic leukemia but also showed persistency and signaling in CD19 CAR T cells that acted as living drugs in two patients with leukemia, a decade after initial treatment, at the expenseof B-cell aplasia due to on-target off-tumor toxicity [6]. Fortunately, B-cell aplasia is a tolerable side effect. However, this is not the case with the on-target off-tumor activity of all CAR T cells [7]. Therefore, the identification of potential target antigens with an ideally minimal expression in normal cells is required to address this issue [8].

Previous studies [9,10] have shown that placental alkaline phosphatase (PLAP) can be a potential antigen for targeted cancer immunotherapy. In fact, PLAP, or alkaline phosphatase, placental type (ALPP), is a glycosylated membrane-bound enzyme that is normally expressed in the placenta. There are also some contradictory reports on the low-level expression of PLAP in normal somatic tissues such as the testis [11,12,13], fallopian tube, uterine cervix, and lung [14,15]. Nonetheless, PLAP has relatively low expression in normal somatic tissues [16], especially when compared to other more studied target antigens in solid tumors, such as Her2 [17] and mesothelin [18,19]. On the other hand, elevated PLAP expression has been reported in several malignancies, including ovarian [9], prostate [10], cervical [20], gastric, pancreatic, urothelial, and colorectal cancers, and testicular seminoma [16,21]. Furthermore, some studies used PLAP-specific monoclonal antibodies [11] and a bispecific T-cell engager [22] to target PLAP on HeLa cervical cancer cells. PLAP-specific CAR T cells have also been successfully used against colon cancer cell lines in cell culture and animal models [23]. These findings suggest that PLAP is a promising target antigen for cancer immunotherapy.

Due to the high expression of PLAP in cervical cancers [14,15] and the importance of cervical cancer as the second most common malignant disease in women [24,25], in this study, we aimed to generate a second-generation PLAP-specific CAR T cell and investigate it against cervical cancer cells. In order to assess the potential tonic signaling in PLAP CAR T cells, we compared their characteristics with CD19 CAR T cells in terms of antigen-independent proliferation, CAR phosphorylation, and differentiation markers. Furthermore, we demonstrated that PLAP CAR T cells could be prospective CAR T cells in cervical cancer cells.

## 2. Materials and Methods

### 2.1. Cell Lines

Plat-A, HeLa, CaSki, and HEK293 cell lines were obtained from the Royan Institute Cell Bank and cultured in Dulbecco’s Modified Eagle Medium (DMEM; GE Healthcare Life Sciences, Pittsburgh, PA, USA) supplemented with 10% heat-inactivated fetal bovine serum (FBS; Thermo Fisher Scientific, Gaithersburg, MD, USA), 1% penicillin/streptomycin (Invitrogen, Eugene, OR, USA), and 2 mM L-GlutaMAX (Thermo Fisher Scientific). All of the cell lines were grown in 85% humidity with 5% carbon dioxide (CO_2_) at 37 °C.

### 2.2. Generation of Retroviral Constructs and Retroviral Vectors

PLAP CAR was designed based on the published sequences of PLAP-specific scFv (B10) [26], human CD8a signal peptide, cMyc-tag, human CD28, and CD3ζ proteins. The sequence was optimized for expression in human cells and then ordered for synthesis. First, the synthetic sequence was cloned into the SFG vector using *Nco*I and *Sph*I (Thermo Fisher Scientific) restriction enzymes. The correct clones were confirmed by sequencing the inserted CAR sequence. Next, to produce the ΔPLAP CAR construct, the signaling portions of CD28 and CD3ζ endodomains were excised using the *Xho*I restriction enzyme, and the plasmid was recirculated by a T4 DNA ligase (Thermo Fisher Scientific). The CAR constructs were transfected into the Plat-A packaging cell line via Lipofectamine 3000 reagent (Thermo Fisher Scientific), and retroviral supernatant was collected at 48 and 72 h post-transfection, then filtered using a 0.45 mm filter and stored at −80 °C.

### 2.3. Generation of CAR T Cells

Written informed consent was collected before isolating peripheral blood mononuclear cells (PBMCs) by ficoll density centrifugation from 3 healthy donor volunteers. The protocols were approved by the Research Ethics Committee at Royan Institute. In a non-tissue culture-treated 24-well plate that had been pre-coated with OKT3 (1 mg/mL) (Ortho Biotech, Inc., Bridgewater, NJ, USA) and CD28 (1 mg/mL) (Becton Dickinson & Co., Mountain View, CA, USA), 1 × 10^6^ PBMCs were seeded into each well. On day one, cells were grown in complete media (RPMI-1640 containing 45% Clicks medium (Irvine Scientific, Inc., Santa Ana, CA, USA), 10% FBS, and 2 mM L-GlutaMAX), which contained recombinant human IL2 (100 U/mL, Royan Biotech, Tehran, Iran). On day 3, after OKT3/CD28 T blast generation, 1 mL of retroviral supernatant was added to a non-tissue culture-treated 24-well plate pre-coated with Retronectin (FN CH-296; Takara Shuzo, Otsu, Japan) and centrifuged for 90 min at 2000× *g*. OKT3/CD28 activated T cells (0.2 × 10^6^/mL) were resuspended in complete media supplemented with IL2 (100 U/mL) and then added to the wells and centrifuged at 400× *g* for 5 min. Transduction efficiency was measured 3 days post-transduction by flow cytometry.

### 2.4. Flow Cytometry Analysis

For analyzing surface markers with flow cytometry, cells were collected, washed, and stained with antibodies (Table 1) at 4 °C for 30 min in the dark. For intracellular staining of phospho-CD3ζ, T cells were fixed with a 1.5% formaldehyde solution (F1635; Sigma-Aldrich, St. Louis, MO, USA), washed, permeabilized with pre-chilled 100% methanol (Fisher Scientific, Pittsburgh, PA) on ice for 15 min, and then washed three times, followed by a CD247(pY142)-specific antibody (Table 1) stain for 60 min at room temperature, in the dark. All samples were acquired on either BD FACSCalibur or BD FACSCanto flow cytometers (BD Biosciences, Franklin Lakes, NJ, USA), and the data were analyzed with the FlowJo software (Tree Star Inc., Ashland, USA).

### 2.5. Cytotoxicity Assay

Luciferase-expressing HeLa or HEK293 cells were seeded into a U-bottomed 96-well plate with D-luciferin (75 μg/mL; Sigma-Aldrich) for cytotoxicity assessment. After that, CAR-expressing T cells were added to the target cells at 20:1, 10:1, 5:1, and 2.5:1 effector: target ratios and incubated at 37 °C in 5% CO_2_ for 2 h. The LUMIstar Omega microplate luminometer (BMG Labtech; Ortenberg, Germany) was used to measure luminescence as relative light units (RLUs). In each assay, for baseline lysis, one group of target cells was cultured alone, and for maximum lysis, another group was cultured in 1% Triton X-100 (Sigma-Aldrich). The percentage of the specific lysis was calculated according to the following formula: % specific lysis = 100 × (spontaneous cell death RLU − sample RLU)/(spontaneous death RLU − maximal killing RLU).

### 2.6. Activation Assessment and Cytokine Measurement

To measure the amount of cytokine expression, 5 × 10^5^ transduced T cells were co-cultured with HeLa target cells at a 1:1 ratio. After 24 h, T cells and media were collected and separated by centrifugation. The cells were subjected to immunostaining with anti-CD25,anti-CD69, and flow cytometry while the supernatants were stored at −20 °C until they were assessed by ELISA or the Luminex assay for detection of secreted cytokines. ELISA assays were performed by the Human Granzyme A DuoSet (DY2905-05, R & D Systems, MN, USA), Human IL-2 DuoSet (DY202, R & D Systems), and Human IFN-gamma DuoSet (DY285B, R & D Systems) as per the manufacturer’s instructions. Human Immunotherapy Luminex Performance Assay 24-plex Fixed Panel (LKTM010, R & D Systems) was used for the Luminex assay.

### 2.7. Co-Culture Experiments

The co-culture of 2 × 10^4^ T cells and 1 × 10^4^ GFP-FFLuc^+^ HeLa cells or CaSki cells (2:1 ratio) was performed in a 12-well plate for 4 (CaSki experiment) or 9 days (HeLa experiment) in 4 mL of complete media, which was not changed during the co-culture. For quantifying cells by flow cytometry, 20 μL of CountBright™ Absolute Counting Beads (C36950; Invitrogen) was added, and 7-AAD was added to exclude the dead cells. The acquisition was halted at 2500 beads.

### 2.8. Statistical Analyses

The statistical significance between groups was determined using a two-way ANOVA followed by Sidak’s multiple comparison test. The *p*-values less than 0.05 were considered statistically significant. Statistical analyses were performed using GraphPad Prism 8.0.2 (GraphPad Software Inc., La Jolla, CA, USA). All results were reported as mean ± SD.

## 3. Results

### 3.1. CAR Construct and its Expression on T Cells

**Figure 1:**
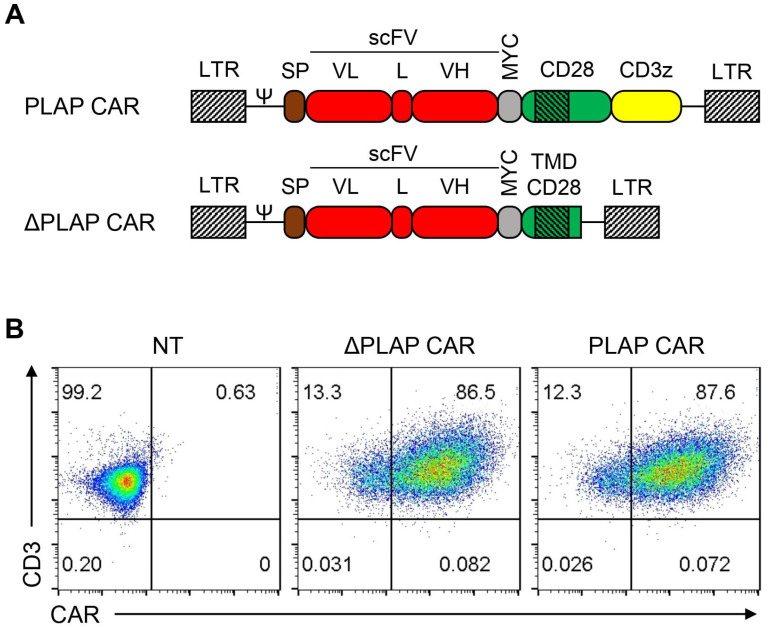
Design and expression of PLAP and ΔPLAP CAR constructs. (**A**) Scheme of PLAP CAR and ΔPLAP CAR. Both CARs contained a signal peptide, a codon-optimized synthetic gene encoding for fully human anti-PLAP scFv, a MYC tag, and the transmembrane region of CD28. Intracellular signaling domains, including CD28 and CD3ζ, are only incorporated into PLAP CAR. (**B**) CAR expression was confirmed on primary T cells by flow cytometry.

We designed a second-generation PLAP CAR containing a CD8α signal peptide, a human scFv against PLAP protein, a cMyc-tag, the CD28 transmembrane, cytosolic domains, and a CD3ζ cytosolic domain. A non-signaling ΔPLAP CAR construct was used as a negative control and differed from PLAP CAR by the lack of CD28 and CD3ζ endodomains (Figure 1A). We checked the expression of CAR molecules on the surface of transduced cells 5–6 days after transduction using an anti-cMYC antibody that binds to the cMYC-tag of PLAP CAR and ΔPLAP CAR. Results showed that typically more than 80% of the transduced T cells expressed PLAP CAR and ΔPLAP CAR on their surfaces (Figure 1B).

### 3.2. Tonic Signaling and Immunophenotype of CAR T Cells

**Figure 2:**
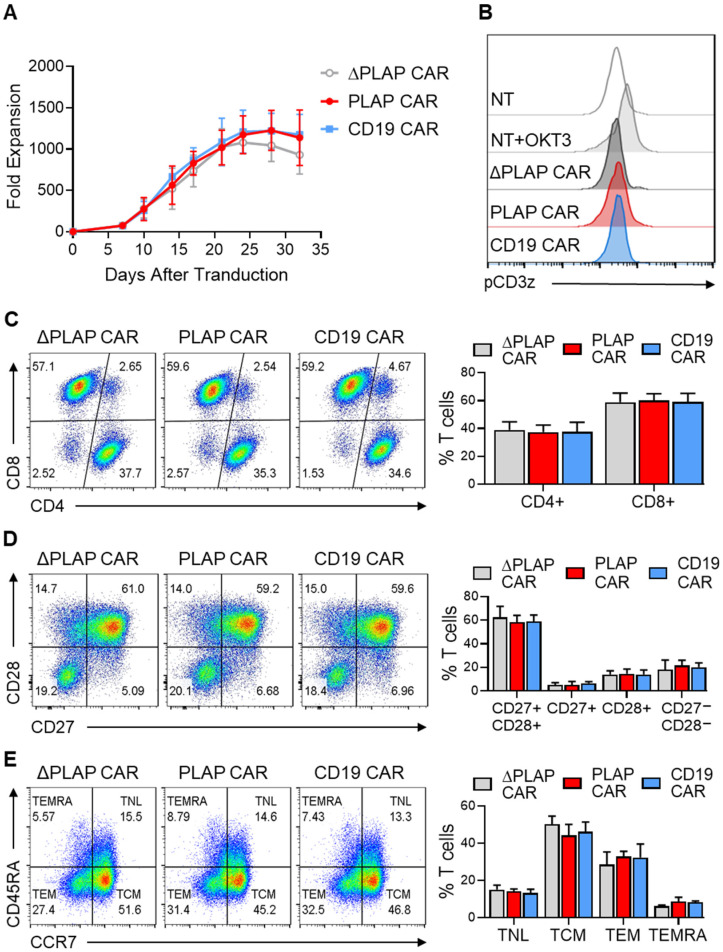
Tonic signaling and immunophenotype characterization of PLAP CAR T cells. (**A**) Absence of tonic signaling was confirmed by evaluating the growth of CAR T cells in the absence of antigen, and (**B**) no independent signaling in signaling domains. Non-transduced (NT) T cells stimulated with OKT3 (anti-CD3) for 24 h were used as a positive control for phosphor-CD3z (pCD3z) staining. (**C**) The ratio of CD4:CD8 among CAR T cells was stable, which was assessed using anti-CD4 and anti-CD8 antibodies by flow cytometry. (**D**) The population of CD27^+^CD28^+^ CAR T cells was higher than previously reported by flow cytometry using anti-CD27 and anti-CD28. (**E**) TCM and TEM have larger populations than TNL and TEMRA, performed by anti-CD45RA and CCR7. TNL is CD45RA^+^CCR7^+^, TCM is CD45^−^CCR7^+^, TEM is CD45RA^−^CCR7^−^, and TEMRA is CD45RA^+^CCR7^−^. *n* = 3.

Since antigen-independent tonic signaling can hamper CAR T-cell anti-tumor activity, we first sought to determine any sign of tonic signaling or adverse effects on the immune phenotype of the expanded CAR T cells. As a positive control, we used a second-generation CD19 CAR containing FMC.63 scFv, which is known to lack significant antigen-independent tonic signaling. We did not detect any antigen-independent growth in PLAP CAR T cells compared with ΔPLAP and CD19 CAR T cells (Figure 2A). Furthermore, we used antibodies against the phosphorylated CD3ζ endodomain in the absence of PLAP antigen to directly assess antigen-independent signaling from the PLAP CAR molecule. According to our findings, ΔPLAP and PLAP CAR T cells, similarly to CD19 CAR T cells and non-transduced T cells, did not phosphorylate in the CD3ζ domain; therefore, CAR T cells did not have tonic signaling (Figure 2B).

We evaluated the CD4:CD8 ratio of CAR T cells by flow cytometry, observing no statistical significance between PLAP CAR T cells and the control groups (Figure 2C). Moreover, we evaluated the expression of CD27 and CD28 costimulatory receptors on CAR T cells which are known to be correlated with T-cell activity and less-differentiated naïve-like and memory phenotypes [27]. Results indicated that the majority (58.4 ± 5.7%) of PLAP CAR T cells were CD27^+^CD28^+^ T cells. More importantly, there was no significant difference in the percentage of CD27 and CD28-expressing T cells between PLAP CAR T cells and the control groups (Figure 2D).

We also evaluated the expression of CD45RA and CCR7 markers on PLAP CAR, ΔPLAP CAR, and CD19 CAR T cells to survey memory differentiation during ex vivo expansion. PLAP CAR T cells contained 14.1 ± 1.3% naïve-like (TNL, CCR7^+^CD45RA^+^), 44.2 ± 6.0% central memory (TCM, CCR7^+^CD45RA^−^), 32.9 ± 2.8% effector memory (TEM, CCR7^−^CD45RA^−^), and 8.7 ± 2.2% CD45RA-expressing effector memory T cells (TEMRA, CCR7^−^CD45RA^+^). We did not detect any significant difference in the percentage of these sub-populations among the experimental groups (Figure 2E).

Overall, these findings suggest that the PLAP CAR construct does not adversely affect the differentiation phenotype of T cells through antigen-independent tonic signaling.

### 3.3. Activation and Functionality of CAR T Cells

**Figure 3:**
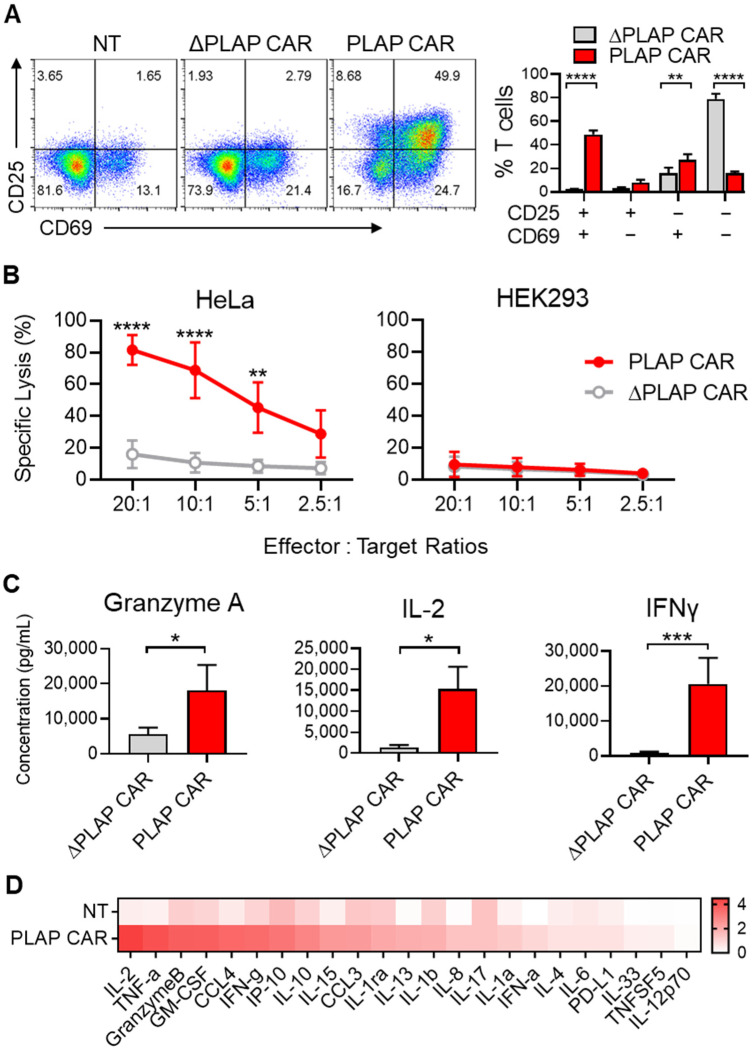
Functional assessment of PLAP CAR T cells. (**A**) The activation markers, such as CD25 and CD69, increased in the nontransduced (NT) and PLAP CAR T-cell population, but the ΔPLAP CAR T-cell population is not a double positive. These markers are quantified with anti-CD25 and anti-CD69 by flow cytometry. (**B**) Luciferase-expressing HeLa or HEK293 cells were co-cultured with PLAP CAR T cells and ΔPLAP CAR T cells, and by increasing the PLAP CAR T cells, the specific lysis was increased. (**C**) Secretory factors, including granzyme A, IL-2, and IFN-γ, were measured by ELISA from the supernatant and PLAP CAR T cells released higher amounts of anti-tumor molecules than ΔPLAP CAR T cells. (**D**) We detected 45 secretory proteins in the supernatant samples of NT and CAR T cells co-cultured with HeLa cells for 24 h. The heat map values represent the mean of three technical replicates. *n* = 3. * *p* < 0.05; ** *p* < 0.01; *** *p* < 0.001; **** *p* < 0.0001.

To test the functionality of PLAP CAR T cells, we evaluated the expression of CD69 and CD25 activation markers on them upon exposure to PLAP-expressing HeLa cells for 24 h. PLAP CAR T cells showed a significant increase in the expression of CD69 and CD25 activation markers compared with the control ΔPLAP CAR T cells (Figure 3A).

Furthermore, by co-culturing HeLa cells with different ratios of PLAP CAR T cells and ΔPLAP CAR T cells, we found that, by increasing the number of PLAP CAR T cells, the specific lysis also increased (20:1 ratio). We observed about 80% specific lysis within two hours. As a control, we used HEK293 cells, which do not express PLAP. We did not observe any significant lysis of HEK293 cells. This demonstrates that the PLAP CAR T cells specifically detect the PLAP antigen expressed on HeLa cells. We also investigated the cytotoxicity of PLAP CAR T cells on CaSki cells, another cervical cancer cell line that reportedly expresses PLAP [28]. The results showed that PLAP CAR T cells significantly decreased the CasKi cell number compared with non-transduced T cells (Figure S1).

To evaluate cytokine production by CAR T cells upon activation, we harvested the supernatant 24 h after co-culture of ΔPLAP CAR or PLAP CAR T cells with HeLa cells and measured the granzyme A, IL-2, and IFN-γ secreted into the media. All three cytokines were secreted at higher levels by PLAP CAR T cells compared to ΔPLAP CAR T cells (Figure 3C). We could also detect several secretory molecules, such as TNF-α, granzyme B, GM-CSF, CCL4, etc. (Figure 3D), in the supernatant of the stimulated PLAP CAR T cells.

### 3.4. Anti-Tumor Activity of PLAP CAR T Cells

**Figure 4:**
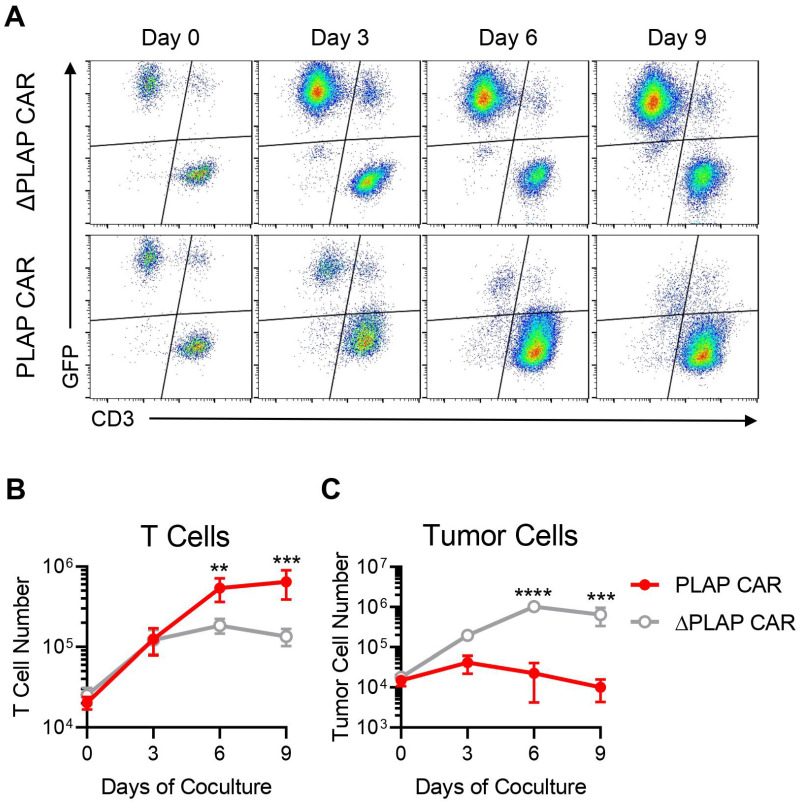
Anti-tumor response of PLAP CAR T cells, in vitro co-culture experiment. (**A**) PLAP CAR T cells and ΔPLAP CAR T cells were co-cultured with HeLa cells, which were indicated by a GFP signal, for 9 days. Cells were collected every 3 days and counted by flow cytometry. PLAP CAR T cells, in comparison with ΔPLAP CAR T cells, demonstrate significant anti-tumor potential, and during 9 days, most of the HeLa cells can be eliminated. (**B**,**C**) These graphs show the population of T cells and tumor cells during co-culture; (**B**) PLAP CAR T cells proliferated much more than ΔPLAP CAR T cells, and in (**C**), the number of HeLa cells, which was eliminated in the presence of PLAP CAR T cells, was significantly higher than when they were co-cultured with ΔPLAP CAR T cells. *n* = 3. ** *p* < 0.01; *** *p* < 0.001; **** *p* < 0.0001.

In order to investigate the anti-tumor capacity of PLAP CAR T cells, we co-cultured the PLAP CAR or ΔPLAP CAR T cells with GFP^+^ HeLa cells for 9 days. During the course of co-culture, PLAP CAR T cells expanded more than 30-fold, reaching around 6.4 × 10^5^ cells on day 9, significantly higher than the number of ΔPLAP CAR T cells; which was around 1.3 × 10^5^ on day 9 (Figure 4A,B). More importantly, the number of HeLa cells decreased in the presence of PLAP CAR T cells, while they continued to proliferate in the presence of the control ΔPLAP CAR T cells (Figure 4A,C). A CD3^+^GFP^+^ population was observed in both groups as early as the first few hours of the co-culture. This population most likely contains doublets of T cells engaged with the HeLa cells, as confirmed by back-gaiting analysis (Figure S2). This observation can be due to the presence of the anti-PLAP scFv in both ΔPLAP CAR and PLAP CAR molecules. These data demonstrate the potent in vitro anti-tumor activity and the tumor control ability of PLAP CAR T cells.

## 4. Discussion

We recently proposed that PLAP can be used as a potent tumor antigen for targeting different solid tumors [16]. High–moderate levels of PLAP expression are only detected in placental tissues, while only a weak PLAP expression has been observed in some endocervical, endometrial, and fallopian tube epithelium samples [21]. This limited PLAP expression in somatic tissues can address the on-target and off-tumor cytotoxicity obstacles in CAR T-cell therapy. We designed and constructed a second-generation PLAP-specific CAR and evaluated its functional characteristics by generating PLAP CAR-modified human T cells.

Tonic signaling is a continuous antigen-independent signaling through the CAR molecule, resulting in chronic stimulation and early exhaustion of T cells [29,30]. Signaling through 4-1BB-bearing CARs has been shown to reduce tonic signaling and T-cell exhaustion compared with CD28-containing CARs [31]. However, this advantage is vector-dependent. For instance, it is reported that 4-1BB signaling in CD19 CAR T cells, unlike CD28, produces positive feedback on the gammaretroviral long terminal repeat (LTR) promoter through NF-κB activation, resulting in CAR T cell apoptosis and inefficient anti-tumor performance [32]. Therefore, we chose to use a CD28 signaling domain in our gammaretroviral PLAP CAR construct. The scFv moiety of the CAR molecule can also contribute to tonic signaling through antigen-independent intermolecular interactions [29,33]. For instance, in the GD2 CAR T cells, these interactions activate a transcriptional profile that accounts for the expression of inhibitory receptors, which causes exhaustion and apoptosis of GD2 CAR T cells [31]. Thus, it is crucial to confirm that any new scFv-based CAR does not mediate tonic signaling and the associated adverse effects on T-cell function. As CD19 CAR T cells recently showed satisfactory results in hematopoietic malignancies [34,35], we used them as a standard indicator to compare the PLAP CAR T-cell characteristics.

Another factor that has a substantial role in CAR T cells is the type of scFv that should be humanized instead of murine, because humanized scFv showed lower immunogenicity compared to murine scFv [36,37,38]. Brudno et al. researched CD19 CAR T cells against B-cell lymphoma and found that humanized scFv showed lower neurologic toxicity and cytokine release syndrome (CRS) than murine scFv [39]. Furthermore, one of the drawbacks of murine scFv compared to human scFv is that after injection, they are eliminated by anti-mouse antibodies; this accounts for the lower persistence of CAR T cells [18,40,41,42]. Recently, Li et al. used PLAP CAR T cells containing a humanized scFv against colon cancer cell lines and showed that PLAP CAR T cells could eliminate colorectal cancers using in vitro and in vivo models. In this study, we used a fully human PLAP-specific CAR T cell.

T-cell subtypes can be distinguished by the expression of the range of CD markers on them, and each of these subtypes exhibits different characteristics, such as immediate action but low persistency, and vice versa [43,44,45]. In cancer treatment, the population of T cells with less differentiated characteristics, such as TNL, TSCM, and TCM, have more persistency, which is favorable because, in the case of cancer relapse, these T cells are required as “living drugs” [46,47]. An investigation on TCM confirmed they have a better long-term expansion, persistency, and anti-tumor effect in vivo [48,49,50]. Moreover, according to Figure 2E, the population of TCM is higher than other subtypes of CAR T cells. Another indicator that shows PLAP CAR T cells are less differentiated than other groups is the high expression of CD27 and CD28 on PLAP CAR T cells (Figure 2D), which can stimulate them against cancer cells [51,52].

During the activation of CAR T cells, they release anti-tumor molecules, including interleukins, perforin, granzyme [53,54], and other markers [55]. By harnessing these characteristics, we showed that during co-culture with target cells, PLAP CAR T cells secrete IFN-γ, granzyme A, and IL-2 and express CD25 and CD69 as well as specific lysis of PLAP^+^ cells, confirming the functionality of PLAP CAR T cells (Figure 3). Furthermore, Li et al. observed specific lysis and release of IFN-γ when they co-cultured PLAP CAR T cells with colon cancer cells (PLAP^+^ cells) [23]. However, PLAP CAR T cells need to be evaluated in terms of other anti-tumor factors, including perforin, granzyme B, and other factors, to more easily compare their cytotoxicity and activation with other CAR T-cell therapy studies.

The cytotoxic activity of PLAP CAR T cells was confirmed by co-culturing with target cells in the long term and comparing their killing capacity with that of ΔPLAP CAR T cells. Although the potential of PLAP CAR T cells for tumor regression needs to be evaluated using normal cervical epithelial cells and animal models, another experiment published during our research showed that PLAP CAR T cells in a colon cancer mouse model resulted in tumor regression [23].

## 5. Conclusions

Here, we demonstrate the importance of constructing CAR T cells against novel antigens that can be a potential asset in solid tumor regression. PLAP CAR T cells can be a prospective weapon in targeted cancer immunotherapy, which needs more investigation into other PLAP^+^ cancer cells to find its way into clinical trials.

## Figures and Tables

**Table 1:** Antibodies used for flow cytometry assays.

Target Protein	Clone	Fluorophore	Company	Cat. No.
cMYC	9E10	FITC	Sigma-Aldrich	F2047
CD3	UCHT1	APC-Alexa Fluor 750	Beckman Coulter (Brea, CA, USA)	A66329
CD4	13B82	Krome Orange	Beckman Coulter	A96417
CD8	SK1	Pacific Blue	BioLegend (Fell, Germany)	344718
CD45RA	5H9	APC	BD Biosciences	561210
CCR7	150503	Alexa Flour 700	BD Biosciences	561143
CD27	O323	PE Cy7	BD Biosciences	567290
CD28	CD28.2	PE Cy5	BD Biosciences	561791
CD247(pY142)	K25-407.69	Alexa Fluor 647	BD Biosciences	558489
CD69	TP1.55.3	ECD	Beckman Coulter	6607110
CD25	M-A251	PE Cy5	BD Biosciences	555433

## Data Availability

The data presented in this study are available upon request from the corresponding author.

## Supplementary Materials

The following supporting information can be downloaded at: https://www.mdpi.com/article/10.3390/biom12091296/s1, Figure S1: in vitro cytotoxicity of PLAP CAR T cells on CaSki cells; Figure S2: A representative data from the coculture experiment with HeLa cells.
