# Supplementary material for: Generation and Functional Characterization of PLAP CAR-T Cells against Cervical Cancer Cells
Source: Biomolecules. 2022 Sep 14;12(9):1296. doi: 10.3390/biom12091296 (PMC9496028; doi:10.3390/biom12091296)
Supplement: Supplementary file 1 [file biomolecules-12-01296-s001.zip › biomolecules-1791163-supplementary.pdf]

Supplementary Figures

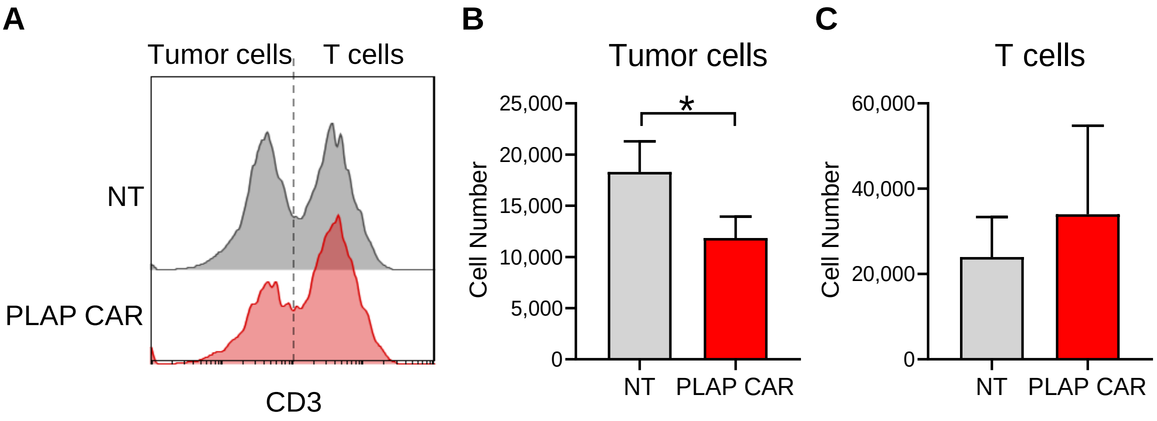

Figure S1. *in vitro* cytotoxicity of PLAP CAR T cells on CaSki cells. (A) PLAP CAR T cells and non-transduced (NT) T cells were cocultured with CaSki cells. Cells were collected after 4 days and counted by flow cytometry using counting beads. Viable T cells and tumor cells were distinguished based on CD3 expression. (B) Decreased number of tumor cells in the PLAP CAR group showed the cytotoxic activity of PLAP CAR T cells. (B) No statistically significant difference was observed in T cells numbers during the 4-day coculture. n = 3, * P < 0.05.

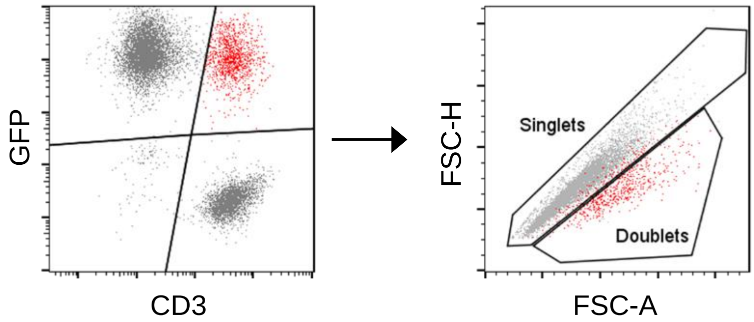

Figure S2. A representative data from the coculture experiment with HeLa cells (Figure 4). Back-gating analysis of the CD3+GFP+ population showed that this population contains doublets, suggesting that CD3+GFP+ events are T cells engaged with the target cells.
